# Operative Techniques to Prevent Dialysis Access-associated Steal Syndrome in High-risk Patients Undergoing Surgery for Hemodialysis Access: A Systematic Review

**DOI:** 10.7759/cureus.6086

**Published:** 2019-11-06

**Authors:** Fareed A Shaikh, Nadeem Siddiqui, Noman Shahzad, Amna Riaz, Ziad Sophie

**Affiliations:** 1 Surgery, Aga Khan University Hospital, Karachi, PAK; 2 Surgery, Northern Lincolnshire and Goole NHS Foundation Trust, Scunthorpe, GBR

**Keywords:** prophylactic intraoperative techniques, high risk patients, dialysis access associated steal syndrome (dass)

## Abstract

Up to 10% of patients suffer from various degrees of dialysis access-associated steal syndrome (DASS) after surgery for hemodialysis access. This systematic review was conducted to find out optimal intra-operative techniques to prevent DASS in high-risk patients.

This systematic review is registered with PROSPERO (2017:CRD42017060804). It was conducted at Department of Surgery, Aga Khan University Hospital, Karachi. All types of studies conducted on intra-operative techniques to prevent DASS in high-risk population (Age > 60 years, female gender, diabetes mellitus, peripheral arterial disease and previous DASS) undergoing access creation from January 1990 till April 2019 were included in the systematic review. Thorough search was conducted on Pubmed, Google Scholar and Cochrane databases to identify relevant articles. Included studies reviewed for success of various techniques to prevent dialysis access steal syndrome are summarized.

Out of 125 studies in the initial search, six met the inclusion criteria. Five were retrospective case series while one was a case report. The largest study sample size was 32. All but one study had arterio-venous access creation on an arm. “Proximalization of arterial inflow” was described in three and “prophylactic distal revascularization and interval ligation (DRIL) procedure” in two studies to prevent DASS. Only one patient out of these studies developed DASS at an overall follow-up of 7-42 months.

Proximalization of inflow has been reported as the most common procedure performed to prevent DASS followed by extension technique and DRIL procedure. All three procedures have satisfactory outcome with no clear superiority of one over the other.

## Introduction and background

End stage renal disease (ESRD) affects more than 1500 people per million population per year. Approximately two-third of these undergo hemodialysis [1]. Hemodialysis access-related complications are the most common cause of hospital admissions in ESRD patients on dialysis [2]. Dialysis access-associated steal syndrome (DASS) is one of those and the most dreadful complication which affects up to 10% of high risk patients [3, 4]. Traditionally, after arterio-venous (AV) access surgery if someone develops DASS, remedial surgery is offered which ranges from salvage procedures usually “distal revascularization and interval ligation - DRIL Procedure” to reciting of AV access. Certain features have been identified that put patients at high risk for developing DASS. These include age more than 60 years, diabetes mellitus, female gender, peripheral arterial disease, and history of previous DASS [3-6]. In these high-risk patients simultaneous prophylactic procedure to prevent DASS along with index AV access surgery for haemodialysis has been reported to reduce the morbidity [7-10]. These prophylactic surgical techniques include “Proximalization of arterial inflow”, “Extension technique” and “Prophylactic DRIL” [7-9].

**Figure 1:**
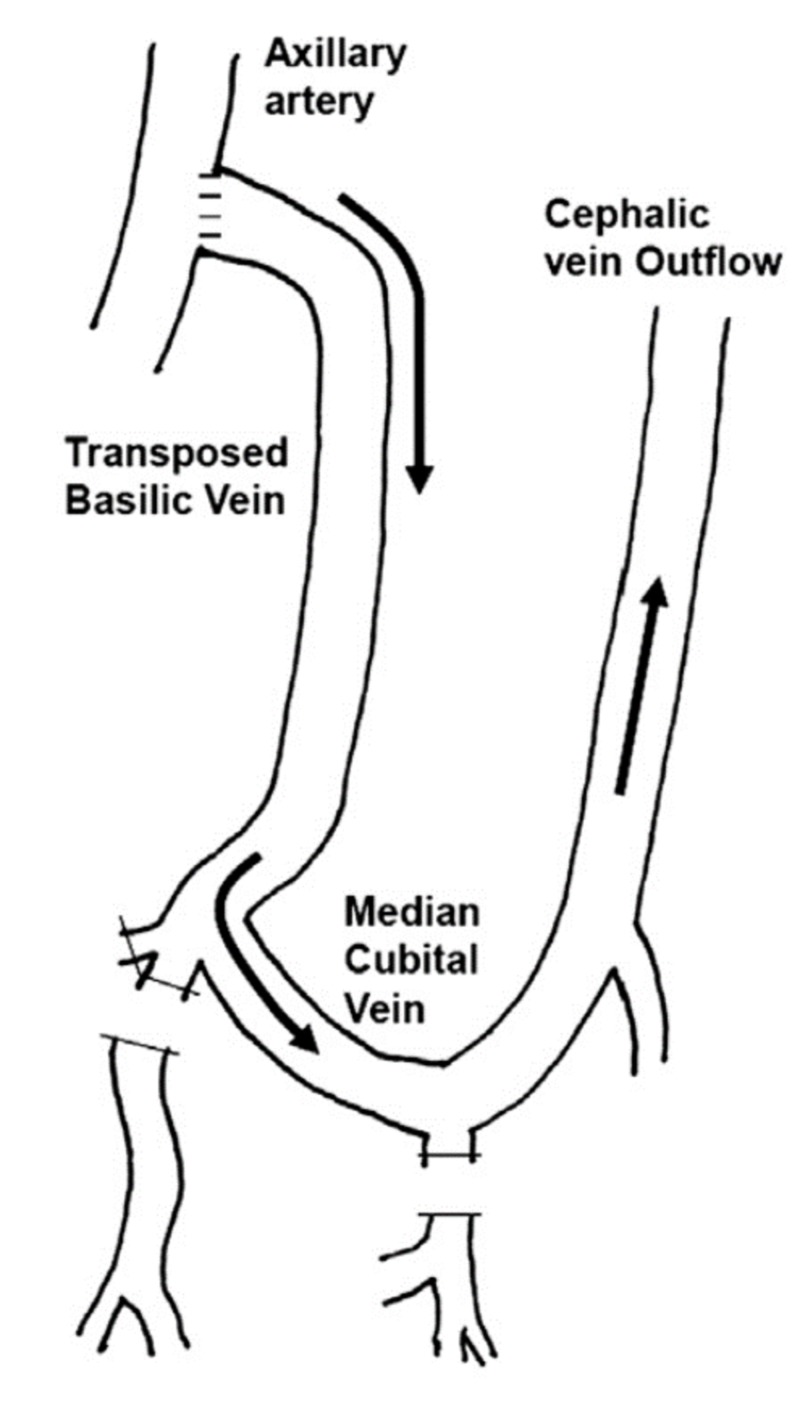
Proximalization of arterial inflow.

Proximalization of arterial inflow as shown in Figure 1 works by taking inflow of the fistula from proximal larger diameter artery (Axillary artery) hence providing adequate blood to both the fistula and distal limb by virtue of higher blood flow [11, 12]. Moreover it also adds to the length of outflow segment and the needling site when reversed basilic vein is used [8]. On the other hand, PTFE graft can also be used, but only at the cost of higher risk of infection and thrombosis [13]. Song and Yun described inflow from sub-scapular artery, which has advantage of proximalization as well as avoiding the main arterial inflow to the limb [13].

**Figure 2:**
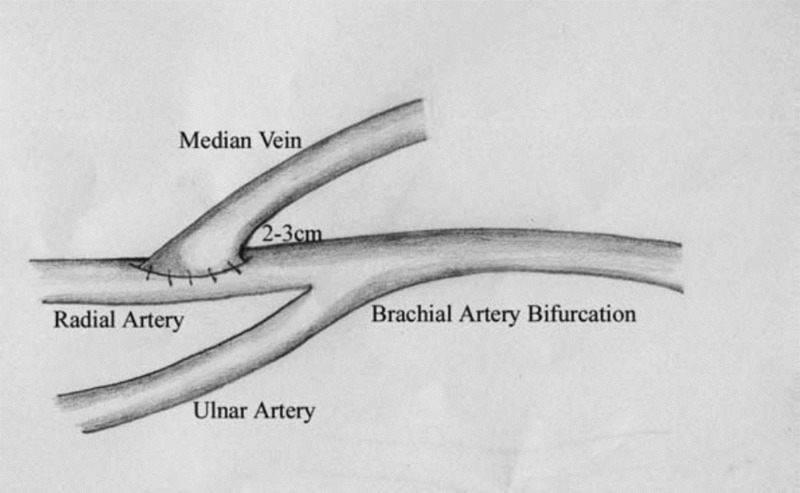
Extension technique.

In the extension technique, instead of taking inflow from brachial artery, one of the forearm arteries is used 2-3 cm distal to bifurcation of brachial artery to anastomose with ante-cubital vein as given in Figure 2 [7]. The perfusion of distal limb is kept intact via the other artery of forearm. It takes 10-15 minutes more than simple brachio-cephalic AV fistula. In addition, it has the additional benefit of maturing both the cephalic and basilic veins simultaneously [7].

**Figure 3:**
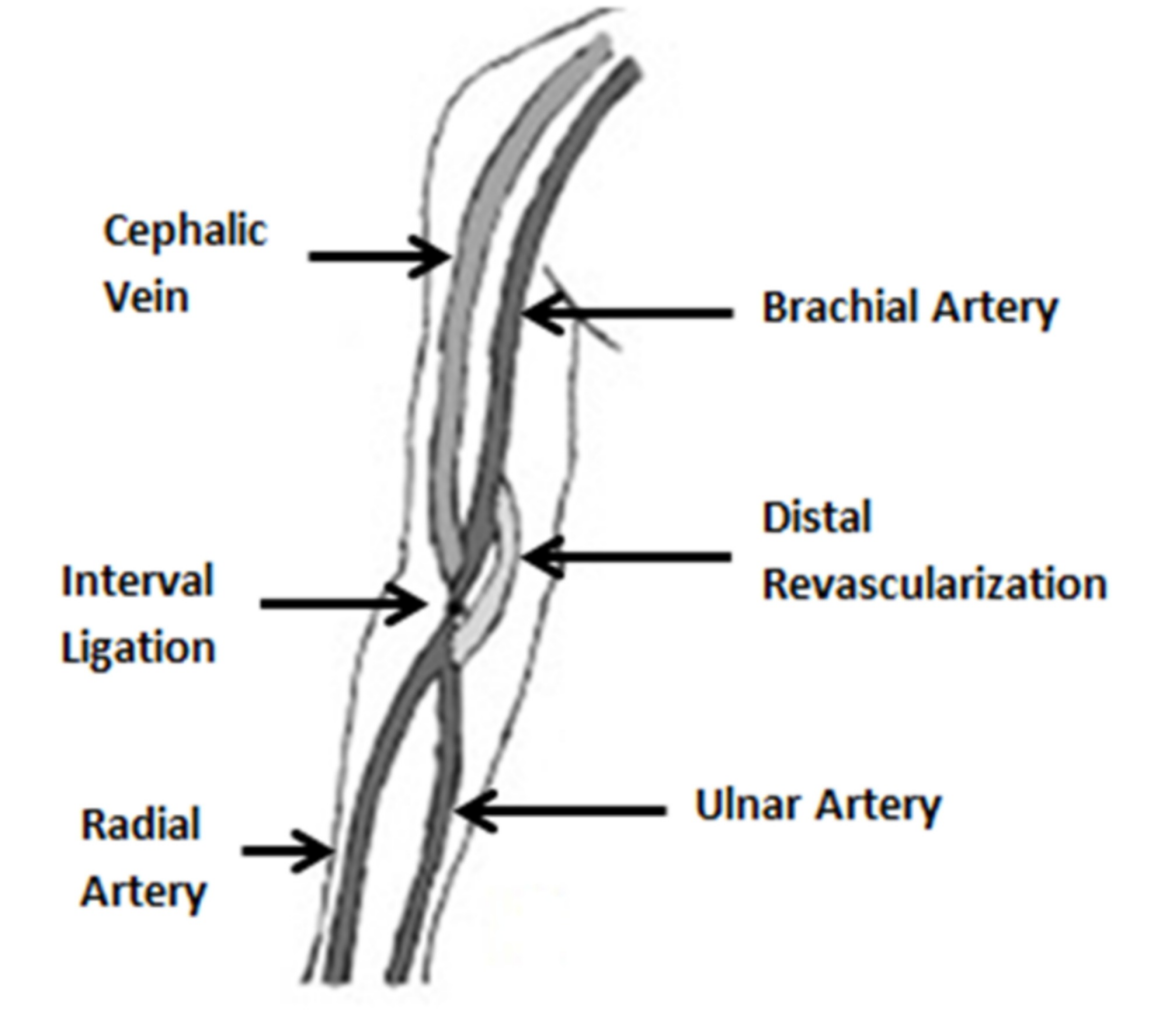
Distal revascularization and interval ligation.

The principle of prophylactic DRIL (Distal Revascularization and Interval Ligation) remains same when it is applied for treatment of DASS, i.e., both the fistula and distal limb are perfused simultaneously and reversal of flow is prevented by interval ligation, as depicted in schematic diagram (Figure 3) [14]. It was first described by Schanzer et al. in 1988 with a technical success of 90% and bypass patency of 80% at four years [15]. Despite all this, one of the major limitations of DRIL procedure is that the native artery is ligated and the limb perfusion is dependent upon bypass graft, which itself is at risk of complications [10]. However, Nader et al. and Leake et al. did not show additional morbidity related to procedure.

Comparative efficacy of these techniques is not known [7-9].

Objective

This systematic review was conducted to review and compare outcome of various prophylactic surgical techniques performed along with primary AV access surgery for hemodialysis to prevent dialysis access steal syndrome (DASS) in high-risk patients.

## Review

This systematic review is registered with PROSPERO (2017:CRD42017060804). We searched for all types of studies including case report, observational studies and interventional trials that reported prophylactic intra-operative techniques to prevent DASS in high-risk patients undergoing hemodialysis access procedure. We considered studies published from January 1990 till March 2017. Studies published in languages other than English, performed on animals or un-published reports were excluded. A thorough systematic search for relevant studies was done on Google scholar, PubMed and Cochrane Database and the last date of search was April 30, 2019.

In order to identify relevant studies, the search strategy was based upon concepts of population, intervention and outcome. Population was identified as adults with end stage renal disease (ESRD), high risk for DASS, and requiring permanent hemodialysis access. Search terms used to look for population of interest were “high risk” OR “prone to” OR “females more than 60 years of age” OR “diabetics” OR “peripheral arterial disease” AND “Arterio-venous fistula” OR “AVF” OR “Hemodialysis Access” OR “AVBG” OR “Brachiocephalic fistula” OR “Basilic transposition fistula” OR “Brachio-basilic fistula”. Intervention of interest was prophylactic intra-operative techniques to prevent DASS for which we used “Prophylactic” OR “Pre-emptive” OR “Preventive” OR “Intra-operative” OR “Per-operative” AND “Techniques” OR “Steps” OR “procedure” OR “Extension technique” OR “DRIL” OR “Proximalization of inflow” OR “RUDI” as relevant terms. Outcome was identified using “Dialysis access steal syndrome” OR “steal syndrome” Or “distal ischemia”.

Using relevant search terms for individual concepts, two investigators separately searched and reviewed the literature. If there was any disagreement between two, the third investigator was involved. Initial screening for inclusion was done reading the title of the studies and identifying duplicates. Further eligibility according to selection criteria was done through stages of abstract and full manuscript evaluation. References of included studies were also searched to identify any missing reports. Specifically designed data extraction sheet was used to collect the data from individual studies. Information such as first author’s name, journal, year of publication, study design, country of origin, target population, prophylactive intra-operative technique used, sample size/number of cases, site of arteriovenous fistula, mean follow-up in months, dialysis access steal syndrome were extracted from studies.

Results

**Figure 4:**
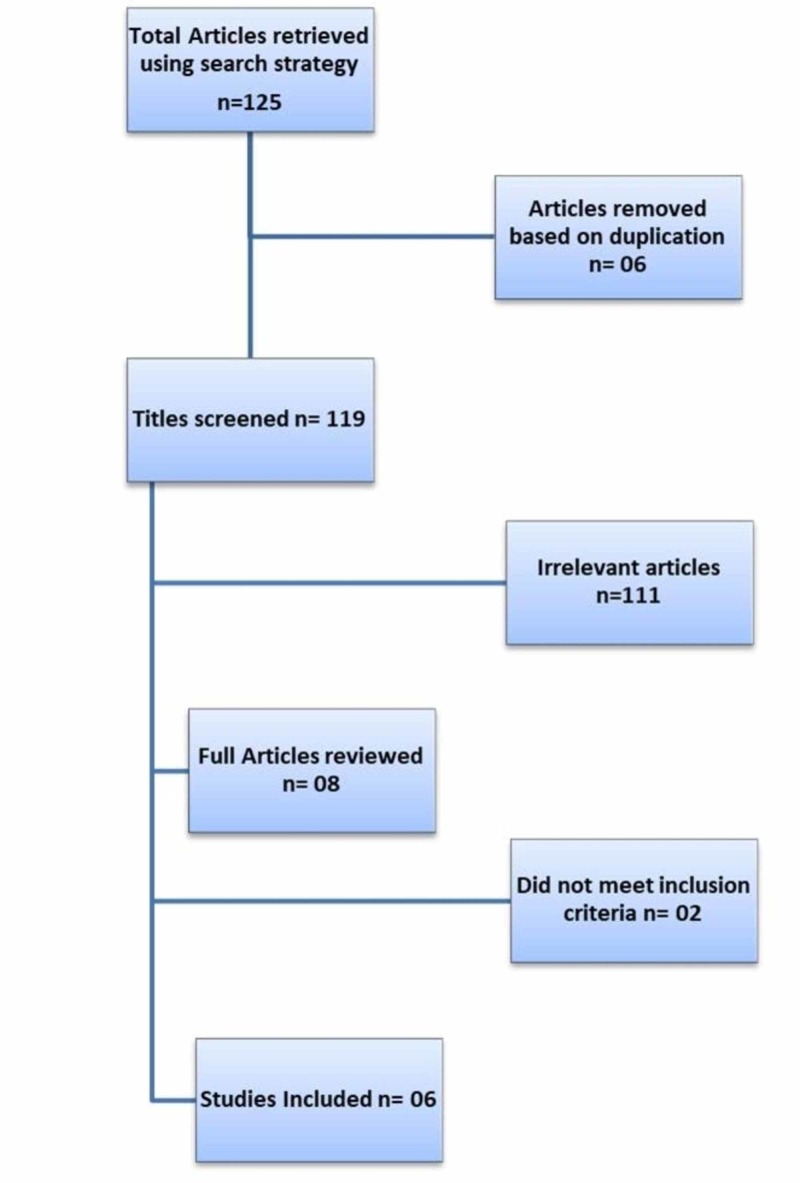
Flow diagram for selection of studies (PRISMA Diagram).

A total of 125 studies were retrieved after applying search strategy, out of those six met the inclusion criteria as summarized in Figure 4.

Majority of studies were retrospective case series while only one was a case report. The largest study sample size was 32 in one of the case series by Ehsan et al. [7]. Five studies involve AV access creation on arm whereas one by Nader et al. has described AV access in thigh [10]. Intra-operative techniques described were variable. “Proximalization of arterial inflow” was described in three, “prophylactic DRIL” in two whereas “Extension technique” was reported in one study to prevent DASS (Table 1).

**Table 1:** Characteristics of the studies included in the systematic review. DASS: Dialysis access-associated steal syndrome; DRIL: Distal revascularization and interval ligation.

Author (Year)	Journal	Study design	Sample size	Population	Intra-operative technique	Country
Ehsan et al. (2005) [7]	Eur J Vasc Endovasc Surg	Case series	32	High risk for DASS	Extension technique	United Kingdom
Jennings et al. (2011) [8]	J Vasc Surg	Case series	04	High risk for DASS	Proximalization of inflow	USA
Jennings et al. (2013) [11]	J Vasc Surg	Retrospective audit	30	High risk for DASS	Proximalization of inflow	USA
Nader et al. (2013) [10]	Ann Vasc Surg	Case series	02	High risk for DASS	Prophylactic DRIL	USA
Song & Yun (2015) [13]	Ann Surg Treat Res	Case report	01	High risk for DASS	Proximalization of inflow (Sub-scapular artery)	Korea
Leake et al. (2015) [9]	J Vasc Surg Cases	Retrospective Case series	05	High risk for DASS	Prophylactic DRIL	USA

**Figure 5:**
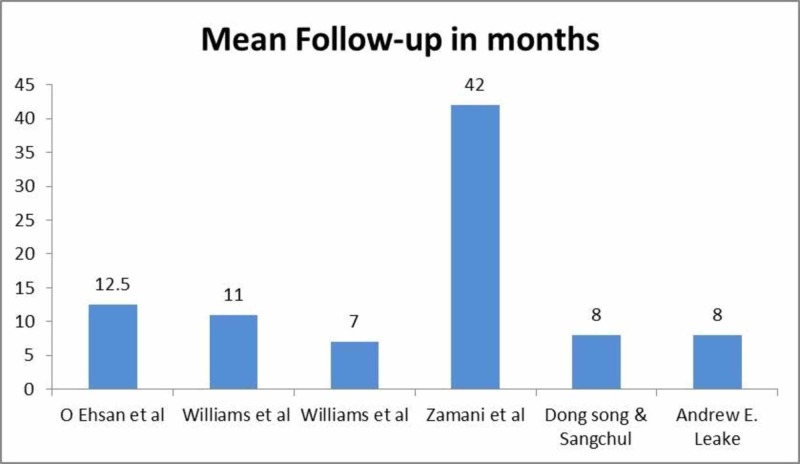
Mean follow-up of patients in included studies.

Follow-up ranged from seven to 42 months as shown in Figure 5. Only one patient in a case series by Ehsan et al. developed DASS which required correction, while none of the patients in other studies developed this complication after prophylactic procedure (Table 2) [7].

**Table 2:** Dialysis access-associated steal syndrome (DASS) in included studies.

Author (Year)	Sample size / No of cases	DASS
Ehsan et al. (2005) [7]	32	01 (3.1%)
Jennings et al. (2011) [8]	04	0
Jennings et al. (2013) [11]	30	0
Nader et al. (2013) [10]	02	0
Song & Yun (2015) [13]	01	0
Leake et al. (2015) [9]	05	0

Our review found that majority of the studies favor “Proximalization of Arterial Inflow” as a useful technique to prevent DASS in high-risk patients undergoing surgery for permanent hemodialysis access, though both “Extension technique” and “Prophylactic DRIL” have also been shown to be effective in preventing DASS. These results can help vascular surgeons in decision making regarding AV access in high risk patients.

Due to lack of large sample studies and retrospective nature of studies, quality of evidence available is low.

## Conclusions

Proximalization of inflow has been reported as the most common procedure performed to prevent DASS followed by extension technique and DRIL procedure. All three procedures have satisfactory outcome with no clear superiority of one over the other.
